# Electroconvulsive Therapy Refusal in Severe Psychosis: Navigating Capacity, Surrogate Decision-Making, and Ethical Duty

**DOI:** 10.7759/cureus.114204

**Published:** 2026-08-09

**Authors:** Karla Perera, Peter Kim, Jessica Barcelo, Domick Cabrera, Jose Cruz

**Affiliations:** 1 College of Medicine, Florida International University, Herbert Wertheim College of Medicine, Miami, USA; 2 Medical School, Nova Southeastern University, Clearwater, USA; 3 Psychiatry, University of Miami Hospital, Miami, USA; 4 Psychiatry, Mount Sinai Medical Center, Miami Beach, USA

**Keywords:** decisional capacity, electroconvulsive therapy (ect), informed consent, involuntary treatment, psychiatric ethics, psychosis, surrogate decision-making, treatment-resistant schizophrenia

## Abstract

Electroconvulsive therapy (ECT) remains among the most effective interventions in psychiatry for severe depression, catatonia, and treatment-resistant psychosis. Despite its well-established efficacy and safety profile, particularly in medically complex and older patients, its use continues to evoke controversy due to the required medical clearance. Ethical dilemmas arise when patients lack insight or capacity to consent, creating tension between respect for autonomy and the duty of beneficence.

We present the case of a 62-year-old woman with a longstanding history of paranoid schizophrenia, general anxiety disorder, hypertension, and hyperlipidemia who was admitted for acute psychosis following medication nonadherence and the recent death of her mother. Her presentation was notable for bizarre delusions, a disorganized thought process, and poor self-care. She was partially compliant with psychotropic therapy, including haloperidol, lorazepam, and lurasidone, but demonstrated limited improvement. Although ECT was considered given the severity and chronicity of her illness, the patient refused treatment. Her family emphasized the gradual deterioration they had observed and advocated strongly for pursuing ECT as the necessary intervention to prevent further decline, given her improvement with past ECT sessions.

Over the course of hospitalization, her symptoms improved only mildly with pharmacotherapy alone, and she remained unable to maintain activities of daily living. This case highlights the enduring clinical and ethical complexities surrounding the use of ECT. It underscores the importance of evaluating capacity, engaging families in shared decision-making, and balancing the principles of autonomy and beneficence in the treatment of severe psychiatric illness.

## Introduction

Electroconvulsive therapy (ECT) continues to be a cornerstone treatment for severe and treatment-resistant mood and psychotic disorders, although it is generally not considered first-line therapy. It is backed by strong evidence for its effectiveness and safety in both acute crises and long-term maintenance [1]. While newer therapies have emerged, ECT remains the only well-established option for achieving rapid symptom relief in life-threatening psychiatric conditions such as catatonia, psychotic depression, and refractory schizophrenia [1].

Clinical and ethical challenges arise when patients lack decisional capacity and refuse ECT, despite the family’s support for ECT and readiness to assume guardianship and decision-making capacity for the patient. The American Psychiatric Association notes that in nonemergent cases, surrogate or court-ordered consent may be necessary, depending on jurisdiction and institutional policy [2]. Ethically, surrogate decision-makers must navigate the tension between respecting the patient’s autonomy and acting in the patient's best interest, particularly given ECT’s potential to restore decisional capacity and significantly improve quality of life [2].

Recent meta-analyses and case series show that ECT is often highly effective in patients without decisional capacity, with response rates reaching up to 80% and many cases resulting in regained capacity to provide informed consent [3-5]. Despite its potential, outcomes are not uniformly favorable. Some patients show only modest clinical gains, remaining functionally impaired and unable to perform basic activities of daily living. These difficult cases underscore the ethical and clinical friction at the heart of involuntary ECT, where the promise of recovery collides with overriding the patient’s autonomy, and families weigh the risks of an invasive procedure performed against the patient’s wishes with the promise of regaining capacity for decision-making, overall clinical improvement, and the patient’s functional ability to live a meaningful life following ECT treatment [5,6].

Despite its proven efficacy, ECT remains underutilized largely due to persistent stigma, fears surrounding cognitive side effects, and strict legal requirements for consent, even in situations where the treatment may be life-saving. The literature emphasizes the critical role of ongoing, iterative consent discussions, thoughtful exploration of the patient’s known values and previously stated preferences, and collaborative, multidisciplinary decision-making. In complex cases involving nonvoluntary ECT, consultation with ethics committees is strongly recommended to ensure that both ethical and clinical standards are upheld [1].

In summary, the clinical and ethical terrain of ECT in severe psychiatric illness is defined by a high-stakes interplay between decisional incapacity, surrogate consent, and unpredictable treatment outcomes. These cases demand more than routine decision-making; they require a rigorous, evidence-based, and ethically sound approach, with clear, defensible documentation at every step. When the patient refuses ECT, it is imperative to obtain collateral information to establish the patient’s baseline and to inform the family of the full risks, benefits, and potential alternatives to treatment with ECT, including the option of no treatment. In these scenarios, it is vital for healthcare providers to not only counsel family members properly to provide truly informed consent, but also to aid them in navigating the underlying ethics involved with the decision. The responsibility falls heavily on the clinical team to navigate these challenges with precision, compassion, and unwavering commitment to patient welfare.

## Case presentation

A 62-year-old Cuban-born woman with a longstanding history of paranoid schizophrenia, hypertension, and hyperlipidemia was brought to the emergency department under an involuntary psychiatric hold due to worsening psychosis, including religiously preoccupied delusions, and inability to care for herself. She resided in an assisted living facility and had a history of medication nonadherence and psychosocial stressors, including the death of her mother approximately six months prior to admission. Her psychiatric illness, diagnosed in 1992, had required multiple past hospitalizations. Shortly after the passing of her mother, she was hospitalized at another facility where treatment with lamotrigine was initiated. She was eventually readmitted to a different facility for Stevens-Johnson syndrome progressing to toxic epidermal necrolysis, requiring ICU management and prolonged recovery. After returning to her assisted living facility, she became increasingly paranoid, isolated, and nonadherent with medications. Her brother, who served as her legal guardian, reported that she had stopped eating, refused visits, and developed fixed delusions, including claiming she was married to another resident and pregnant "because God told her so." Her brother reported that this was a significant decline from the patient’s baseline and that she had a prior history of improving on two separate occasions with ECT.

On arrival at the hospital, she was guarded, minimally cooperative, and refused examination. She spent most of the early hospitalization isolated in bed, with a flat affect, severe avolition, persistent delusions, and profound impairment in insight and judgment. As her symptoms persisted, she was treated with an extensive antipsychotic regimen that included haloperidol 10 mg twice daily (linked to an intramuscular formulation), aripiprazole 5 mg daily (linked to olanzapine 5 mg IM), and chlorpromazine 50 mg IM available three times weekly as needed prior to ECT. She was briefly prescribed lurasidone 40 mg with dinner (a medication taken prior to arrival) but was never compliant, and it was therefore discontinued. Haldol decanoate 150 mg IM was administered three days apart, with 300 mg IM administered 28 days later. She also received lorazepam 1 mg nightly (eventually decreased to 0.5 mg nightly) for anxiety and amlodipine 10 mg daily for hypertension (with which she was intermittently compliant), with PRN clonidine available. Additionally, oral losartan 25 mg was initiated once her compliance improved with ECT. Despite adherence to this multi-agent pharmacologic regimen, she demonstrated minimal improvement, remained socially withdrawn, continued expressing delusional beliefs when engaged, and consistently refused formal psychiatric interviews prior to initiation of ECT.

Given her persistent negative symptoms, severe functional decline, and lack of response to maximal pharmacotherapy, she was evaluated for involuntary treatment. She repeatedly refused discussions about ECT; however, she lacked the capacity for medical decision-making. After psychiatric evaluation and court review, she was formally declared incompetent, and her legal guardian was finally granted the ability to provide consent for ECT.

**Figure 1:**
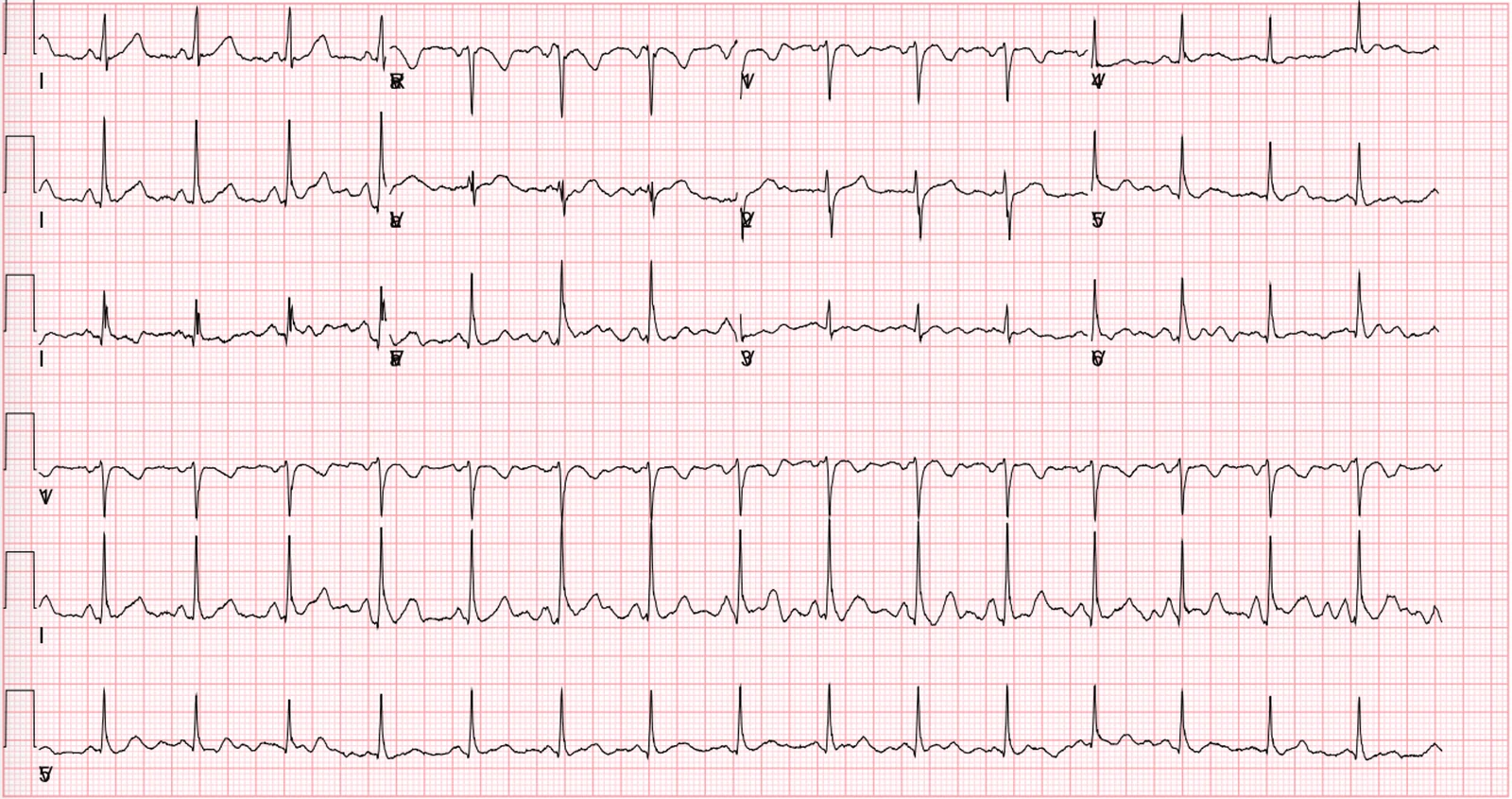
Pre-electroconvulsive therapy 12-lead electrocardiogram. ECG demonstrated a normal sinus rhythm with a QTc of 455 ms.

**Figure 2:**
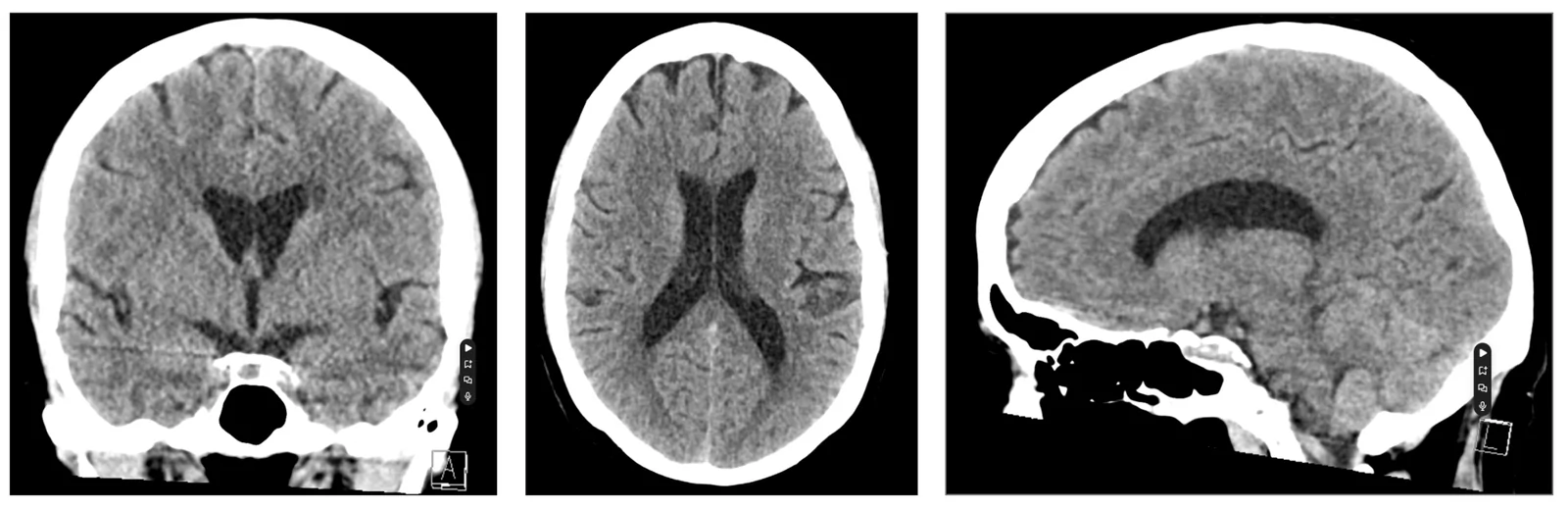
Non-contrast computed tomography images of the head obtained during the pre-electroconvulsive therapy evaluation. Representative (A) coronal, (B) axial, and (C) sagittal images respectively are shown. The imaging revealed no acute intracranial findings.

Mental status examination during this period was limited by her refusal to participate but, when obtainable, revealed a disheveled woman with poor hygiene who often lay in bed covered by a blanket and provided short or hypoverbal replies. She was intermittently alert and oriented but demonstrated a blunted affect, a disorganized thought process, paranoid delusions, and markedly impaired judgment and insight. No abnormal movements or extrapyramidal symptoms were noted. Her cardiopulmonary and abdominal examinations were unremarkable. The findings of all laboratory investigations, including their respective reference ranges, are presented in Table 1. Laboratory studies, including CBC and a basic metabolic panel, were within normal limits except for a low blood urea nitrogen (BUN) of 5 mg/dL, and toxicology screening was negative. The ECG demonstrated a normal sinus rhythm with a QTc of 455 ms, as shown in Figure 1. Representative coronal, axial, and sagittal images from the noncontrast head CT are shown in Figure 2A-C, respectively, and revealed no acute abnormalities. She was medically cleared for ECT. 

**Table 1:** Laboratory investigations obtained during the pre-electroconvulsive therapy evaluation BUN: blood urea nitrogen, eGFR: estimated glomerular filtration rate,nRBC: nucleated red blood cell.

Parameter	Patient Value	Reference Range
Sodium	138 mmol/L	136-145 mmol/L
Potassium	4.1 mmol/L	3.5-5.1 mmol/L
Chloride	101 mmol/L	98-107 mmol/L
Carbon dioxide (CO₂)	24.9 mmol/L	21-32 mmol/L
Glucose	179 mg/dL	74-106 mg/dL
Blood urea nitrogen (BUN)	5 mg/dL	7-18 mg/dL
Creatinine	0.57 mg/dL	0.55-1.02 mg/dL
Calcium	9.3 mg/dL	8.5-10.1 mg/dL
Anion gap	12.1 mmol/L	5-15 mmol/L
BUN/creatinine ratio	8.8	8.0-30.0
Calculated osmolality	288 mOsm/kg	275-295 mOsm/kg
Estimated glomerular filtration rate (eGFR)	103 mL/min/1.73 m²	≥60 mL/min/1.73 m²
Lymphocytes (%)	14.6%	16.0-45.0%
Monocytes (%)	8.8%	2.0-12.0%
Eosinophils (%)	0.9%	0-5%
Basophils (%)	0.2%	0-2%
Nucleated red blood cells (nRBC, %)	0.0%	0.0-0.2%
Immature granulocytes (%)	0.40%	0.00-0.50%
Absolute neutrophil count	6.98 × 10³/µL	1.80-7.20 × 10³/µL
Absolute lymphocyte count	1.36 × 10³/µL	1.20-4.00 × 10³/µL
Absolute monocyte count	0.82 × 10³/µL	0.20-1.00 × 10³/µL
Absolute eosinophil count	0.08 × 10³/µL	0.00-0.45 × 10³/µL
Absolute basophil count	0.02 × 10³/µL	0.00-0.20 × 10³/µL
Absolute nucleated red blood cell count	0.000 × 10³/µL	0.000-0.012 × 10³/µL
Absolute immature granulocyte count	0.04 × 10³/µL	0.00-0.03 × 10³/µL

She underwent a series of 12 bilateral ECT treatments administered under general anesthesia. Treatment parameters included electrical charges between 192 and 224 millicoulombs, energies of 33 to 47.7 joules, pulse widths of 0.5 milliseconds, frequencies between 30 and 85 hertz, motor seizure durations of 37 to 47 seconds, and seizure durations ranging from 52 to 81 seconds. Dynamic impedance consistently ranged from 217 to 249 ohms. She tolerated all treatments without complications, including the absence of postictal agitation, confusion, or delirium. Nursing staff noted progressive improvement in her cooperation during transport to and from treatment.

Over the course of ECT, gradual but meaningful clinical improvement emerged. Her affect brightened, and her paranoia diminished. Her speech became more fluent, her thought process more linear and coherent, and she engaged more spontaneously with staff. Following completion of the twelfth treatment, she appeared significantly closer to her reported baseline: calm, cooperative, alert, and oriented, without acute distress. She denied hallucinations, paranoia, and suicidal or homicidal ideation. Compared to her pre-ECT presentation, she demonstrated substantial improvement in functional status, motivation, and cognitive organization, consistent with a favorable therapeutic response to ECT after failure of extensive antipsychotic therapy.

## Discussion

This patient’s presentation was consistent with an acute exacerbation of chronic paranoid schizophrenia, precipitated by medication nonadherence and psychosocial stressors. Her persistent delusions, disorganized thought process, severe avolition, and inability to perform basic activities of daily living demonstrated a high burden of illness and clear functional decline. The diagnostic picture, coupled with failure to respond to an extensive antipsychotic regimen, including haloperidol, aripiprazole, and chlorpromazine (only administered twice, as needed prior to the first ECT session), supported the medical justification for ECT as the next-line intervention for treatment-resistant schizophrenia [1]. ECT is well established as an effective therapy for refractory psychosis, particularly in older adults and medically complex patients, and it remains the only modality capable of producing rapid symptomatic improvement when pharmacologic interventions fail [1].

A central challenge in this case was the patient’s absence of decisional capacity. Throughout hospitalization, she was unable to understand, appreciate, or rationally process information regarding her illness or proposed treatments. She refused interviews, rejected discussions of ECT, and was unable to articulate risks or benefits in a coherent manner. Decisional incapacity in severe psychosis is common and fluctuates, but in this patient, it was persistent and functionally impairing, leaving her incapable of informed refusal [3,4]. This created a clinical situation in which autonomy could not be meaningfully expressed, requiring the treatment team to rely on statutory mechanisms for surrogate consent [3,5,6].

The legal dimension of this case was therefore central. After formal assessment and documentation of incapacity, the court appointed a health surrogate to provide consent for treatment, consistent with state statutes governing psychiatric care for incompetent adults. Surrogate decision-making in psychiatry is ethically complex because it introduces a proxy who must balance substituted judgment (what the patient would want) with the patient's best interest (what promotes the patient’s welfare) [3,5,6]. In this instance, the patient's brother provided extensive collateral describing months of decline, refusal of care, and functional collapse, and he expressed consistent concern regarding the trajectory of her illness. Based on prior outpatient discussions about ECT and the desire to improve the condition of the patient, the brother advocated for ECT as a necessity and considered it an appropriate intervention for the patient. Unlike cases in which families resist guardianship or oppose ECT out of fear, stigma, or strained relationships, this surrogate accepted legal responsibility and endorsed ECT after observing the patient’s continued deterioration and lack of improvement with medication.

Ethically, this case reflects a resolution, rather than a conflict, between respect for autonomy and the duty of beneficence. Autonomy is a core bioethical principle, but its application presumes decisional capacity. When psychiatric illness erodes the cognitive and volitional foundations of autonomy, clinicians face the obligation to act beneficently to prevent further harm, even when this requires overriding contemporaneous refusal [3,5,6]. Involuntary ECT remains controversial precisely because of this tension [5,6]. Critics argue that overriding refusal risks unjustified paternalism, while proponents contend that beneficence and nonmaleficence require clinicians to intervene when untreated psychosis poses substantial and ongoing harm [5,6]. The decision made reflects a convergence of family advocacy, clinical judgment, and appropriate legal safeguards. The ethical justification in this case rests on three pillars: (1) clear and documented incapacity, (2) failure of intensive pharmacologic therapy, and (3) surrogate consent obtained through a legally validated process [3,5,6].

Another essential dimension is the role of collateral information, which shaped both the diagnostic understanding and the ethical-legal framework. The brother’s account of the patient’s precipitous functional decline, refusal to eat, inability to maintain hygiene, and fixed delusions established the severity and chronicity of impairment beyond what was visible during inpatient interactions alone. Collateral thus became not merely clinically informative but ethically necessary, guiding the determination of incapacity and supporting the surrogate’s decision that ECT aligned with the patient’s best interests. In psychiatric ethics, collateral input often bridges the gap between patient autonomy and real-world functioning, especially when insight is profoundly impaired.

Finally, the patient’s outcome reinforces the clinical and ethical rationale for pursuing ECT with surrogate consent. Over 12 treatments, she demonstrated significant improvement in affect, engagement, thought organization, and overall functioning. Although ECT does not guarantee remission, her substantial clinical gains underscore that beneficence-driven intervention can restore capacities compromised by illness, potentially re-enabling autonomy that would otherwise remain inaccessible [3-5]. This outcome illustrates an important ethical principle: that temporary suspension of autonomy via surrogate consent may ultimately serve autonomy by treating the illness that impairs it [3,5,6].

Overall, this case exemplifies the intricate interplay between diagnosis, treatment resistance, legal structures, surrogate decision-making, and core ethical principles in psychiatry. It highlights the necessity of thorough capacity evaluation, structured collateral gathering, clear documentation, and continuous ethical reflection when considering ECT in patients unable to provide informed consent [3,5,6]. These cases occupy one of the most challenging intersections of psychiatry, where clinicians must navigate law, ethics, family dynamics, and clinical urgency to determine the most justifiable and humane course of action [3,5,6].

## Conclusions

In summary, this case illustrates the challenging intersection of clinical urgency, legal constraints, and ethical decision-making that arises when patients with severe psychosis lack capacity and refuse treatment. With thorough assessment, clear communication with surrogates, and adherence to established ethical frameworks, ECT can be administered responsibly and effectively, even in the context of involuntary treatment. The patient’s substantial improvement underscores the therapeutic potential of ECT in restoring functioning and autonomy when pharmacologic options have been exhausted.

## References

[1] Espinoza RT, Kellner CH (2022). Electroconvulsive therapy. N Engl J Med.

[2] Wilson JE, Oldham MA, Francis A (2025). Catatonia: American Psychiatric Association resource document. J Acad Consult Liaison Psychiatry.

[3] Takamiya A, Sienaert P, Gergel T, Gather J, Kishimoto T, Zilles-Wegner D (2022). Effectiveness of electroconvulsive therapy in patients lacking decision making capacity: a systematic review and meta-analysis. Brain Stimul.

[4] Tor PC, Tan FJ, Martin D, Loo C (2020). Outcomes in patients with and without capacity in electroconvulsive therapy. J Affect Disord.

[5] Besse M, Methfessel I, Simon A, Wille C, Zilles D (2019). Electroconvulsive therapy in incapable patients refusing treatment: prevalence, effectiveness, and associated factors. J ECT.

[6] Methfessel I, Sartorius A, Zilles D (2018). Electroconvulsive therapy against the patients' will: a case series. World J Biol Psychiatry.

